# Inherited gastrointestinal stromal tumor syndromes: mutations, clinical features, and therapeutic implications

**DOI:** 10.1186/2045-3329-2-16

**Published:** 2012-10-04

**Authors:** Michael A Postow, Mark E Robson

**Affiliations:** 1 Department of Medicine, Memorial Sloan-Kettering Cancer Center, New York, NY, USA; 2 Clinical Genetics and Breast Cancer Medicine Services, Memorial Sloan-Kettering Cancer Center, New York, NY, USA; 3 Weill Cornell Medical College of Cornell University, New York, NY, USA

**Keywords:** Gastrointestinal stromal tumor, c-KIT, Platelet-derived growth factor-alpha, Neurofibromatosis, Carney triad, Carney-stratakis syndrome, Succinate dehydrogenase

## Abstract

The discovery of underlying molecular genetic abnormalities in gastrointestinal stromal tumors (GISTs) such as activating mutations in the tyrosine kinase genes, *KIT* and *platelet derived growth factor receptor-alpha (PDGFRA),* has led to remarkable clinical advances in treatment. Small molecule inhibitors such as imatinib and sunitinib are known to inhibit the aberrantly activated KIT and PDGFRA receptor signaling and can lead to excellent clinical outcomes for patients with GIST. Though the majority of GISTs appear to arise sporadically, a number of families with high frequencies of GISTs have been reported and germline mutations have been identified. This review will highlight the various inherited mutations associated with familial GIST syndromes and describe how an improved understanding of these genetic syndromes has important clinical implications for future understanding of this heterogeneous disease.

## Introduction

Gastrointestinal stromal tumors (GISTs) are the most common mesenchymal tumors arising in the gastrointestinal tract [1,2]. Though the majority of GISTs appear to arise sporadically, a number of families with high frequencies of GISTs have been reported and germline mutations have been identified [3]. The true frequency of all GIST diagnoses has been difficult to determine because the definition of GIST was derived in 1990 before it was molecularly characterized. One United States report from the Surveillance, Epidemiology, and End Results (SEER) database indicated that, from 1992 to 2000, the yearly incidence rate in the United States was 6.8 cases per million [4]. The reported US annual incidence rate is slightly lower than incidence rates reported in several international epidemiological studies with highest described incidence of 14.5 cases per million in Sweden [5-7]. Discrepancies between diagnostic criteria over the time period of data collection may have accounted for some of the variation. No specific epidemiological risk factors for GIST have been described.

The majority of GISTs appear to be sporadic, but a number of families with inherited predisposition to GISTs have been identified. The first family with features consistent with inherited GIST was reported in 1990, but it was not until 1998 that Nishida and colleagues identified the first germline mutation associated with familial predisposition to GIST [8,9]. In this Japanese family, three individuals in two generations were diagnosed with multiple GISTs. The germline DNA of the available affected family members contained a mutation in exon 11 of *c-KIT*, which resulted in deletion of a valine residue at codon 559_560 in the juxta-membrane domain of the KIT protein. This same mutation was observed in the subjects’ GIST tumors and resulted in constitutive activation of KIT.

Since this first description of a family with an exon 11 *KIT* mutation, multiple other families with inherited GIST syndromes have been described. Though many have been found to have exon 11 *KIT* mutations, others have alternative *KIT* mutations or mutations involving *PDGFRA, neurofibromatosis- 1 (NF1),* and *succinate dehydrogenase (SDH*) genes [10-29]. Similarities exist among the clinical features of these various germline familial GIST mutations, but each germline mutation can manifest differently (Table 1) .

**Table 1:** Germline Mutations Associated with GIST Predisposition and Associated Clinical Features

**Gene (Exon)**	**Mutation**	**General Clinical Features**	**Reference**
***C-KIT*(11)**	V559del	Hyperpigmentation, urticaria pigmentosa, dysphagia	Nishida 1998 [9]
	CAACTTdup		Carballo 2005 [11]
	V559A		Beghini 2001 [30]
	W557R		Maeyama 2001 [31]
			Robson 2004 [26]
***C-KIT*(17)**	D820Y	Dysphagia but no hyperpigmentation	Hirota 2002 [32]
***C-KIT*(13)**	K642E	No hyperpigmentation and no urticaria pigmentosa	Isozaki 2000 [33]
			Graham 2007 [34]
PDGFRA*	D846Y	Large hands	Chompret 2004 [35]
	Y555C	Lipomas and small intestine fibrous tumors	de Raedt 2006 [36]
	V561D		Pasini 2007 [37]
NF1**	Many various mutations in *NF1* gene (>300 identified)	Café au lait spots, dermal neurofibromas, axillary/inguinal freckling, ocular hamartomas	Relles 2010 [38]
SDHB, SDHC, and SDHD***	*SDHB* IVS1 + 1 G → T	Paraganglioma and pheochromocytoma	Carney 2002 [39]
	*SDHB* c.423 + 1 G → C	(Carney-Stratakis Syndrome)	McWhinney 2007 [40]
	*SDHB* c.45_46insCC		
	*SDHC* c.43 + 1 C → T		
	*SDHC* IVS5 + 1 G → A		
	*SDHD* c.57delG		

*Platelet derived growth factor receptor-alpha.

**Neurofibromin 1.

***Succinate dehydrogenase (B, C, and D).

## C-kit mutations

Paralleling the high frequency of KIT mutations in sporadic GIST, most reported inherited GISTs have involved families with germline mutations in the *KIT* gene, commonly in exon 11, which encodes the juxta-membrane domain. In addition to the development of GISTs, these families manifest variable clinical phenotypes that typically also include hyperpigmentation, urticaria pigmentosa, and dysphagia. The specific *KIT* exon 11 deletion mutation described in the first family with an identified germline mutation in familial GIST, does not seem to be required for the familial GIST syndrome as a similar clinical phenotype involving hyperpigmentation and GIST predisposition was seen in a Spanish family with an alternative exon 11 *KIT* mutation consisting of a duplication of the sequence CAACTT [30].

Missense mutations involving exon 11 have been implicated in similar familial GIST syndromes. Several families have to found with germline point mutations leading to the substitution of alanine for valine (559) in the KIT juxta-membrane domain [31]. Another exon 11 *KIT* juxta-membrane missense mutation (W557R) was described in a family consisting of 19 individuals of Italian ancestry who had variable expression of clinical phenotype involving hyperpigmentation and dysphagia [26]. Although the development of GIST was nearly uniform in this kindred, not all family members harboring this specific germline mutation had hyperpigmentation or dysphagia, suggesting a degree of variability of expression, even within a family with a specific KIT germline mutation.

*KIT* exon 11 mutations are not the only *KIT* mutations implicated in familial GIST syndromes. Hirota *et al.* (2002) reported the first identified family with a germline mutation in exon 17, encoding the KIT tyrosine kinase II domain [32]. Though the precise clinical phenotype of *KIT* mutations may be influenced by reporting, none of the members of this family with KIT tyrosine kinase II domain mutations had hyperpigmentation, differing from some families with *KIT* exon 11 mutations. Dysphagia, however, was a common similar complaint and suggests dysphagia may be a feature more characteristic of germline KIT mutations in general, rather than associated with a specific mutation.

A third KIT mutation in exon 13 encoding the tyrosine kinase I domain, has also been implicated in familial GIST syndrome. Families have been described that have single base mutations in the tyrosine kinase I domain resulting in a substitution of Glu for Lys (642) [33,34]. A predisposition to GIST was present in these families but not hyperpigmentation or urticaria pigmentosa, providing further support to the fact that specific *KIT* germline mutations may lead to variable clinical phenotypes.

Mouse models with “knock-in” mutations, representing inherited GIST syndromes, additionally support the slightly different clinical phenotypes associated with specific *KIT* mutations. Mice with the V558del mutation (corresponding to the human exon 11 deletion mutation) as well as the D818Y mutation (corresponding to human exon 17 missense mutation) had interstitial cell of Cajal hyperplasia and GISTs [41,42]. Only mice with V558del had increased dermal mast cells, a finding not seen in mice with the D818Y mutation. This suggests the specific KIT exon 11 mutation may be required for the feature of urticaria pigmentosa whereas development of GIST may be a more generalized phenomenon, associated with a broad spectrum of KIT activating germline mutations. Despite the suggestion of genotype specific clinical features, variability in patient reporting of additional components of the inherited GIST syndrome may complicate precise genotype-phenotype correlations as suggested in one report [26].

## PDGFRA mutations

Though the majority of described inherited GIST syndromes have been associated with germline *KIT* mutations, several families with inherited predisposition to GISTs have been described with germline *PDGFRA* mutations. One French family with five affected individuals was found to have a germline *PDGFRA* missense mutation (2675 G > T), resulting in a tyrosine substitution for the highly conserved aspartic amino acid at codon 846 which showed perfect cosegregation with the GIST phenotype in the tested family members [35]. Affected individuals also had large hands whereas those without the mutation, did not. Interestingly, the PDGFRA Asp846Tyr mutation identified in this family is homologous to codon 820, located on the KIT tyrosine kinase II domain, the site of inherited GIST involving a Japanese kindred of six affected family members [32].

Other *PDGFRA* mutations, in addition to Asp846Tyr, have been associated with inherited GIST. Three sisters who were affected with intestinal neurofibromatosis in an autosomal dominant pattern of inheritance, without other manifestations of neurofibromatosis 1 (NF1) or neurofibromatosis 2 (NF2), underwent genetic screening, and a PDGFRA Y555C mutation, located in the juxta-membrane domain, was identified [36]. The similarity of these tumors’ genetic composition and clinical phenotype to GIST led the authors to conclude that intestinal neurofibromatosis is a specific subtype of KIT negative, familial GIST, associated with PDGFRA mutations and not a completely distinct disease. Affected individuals had large hands, a finding similar to other families described with inherited GIST and PDGFRA mutations, though not described in familial GIST associated with *KIT* germline mutations. None of the other related clinical manifestations of inherited GIST (dysphagia, hyperpigmentation, urticaria pigmentosa) associated with germline *KIT* mutations was seen in patients with PDGFRA mutations.

A third *PDGFRA* germline missense mutation (V561D) involving exon 12 was identified in a young female patient with several gastric GISTs [37]. This patient’s alternative germline PDGFRA mutation may have resulted in her slightly different phenotype as she was noted to have, in addition to gastric GISTs, multiple lipomas and fibrous tumors of her small intestine. Lipomas and fibrous tumors of the small intestine were not previously part of other families described [35,36] with PDGFRA mutations and predisposition to GIST nor where they a feature of families with *KIT* germline mutations.

## Neurofibromatosis type 1; von Recklinghausen’s disease (NF1)

NF1 is a common genetic disorder occurring in approximately 1 in every 3,000 live births. The disease is classically associated with café au lait spots, multiple dermal neurofibromas, axillary and inguinal freckling, and ocular hamartomas [43]. NF1 is inherited in an autosomal dominant manner, and the mutated NF1 protein encodes the GTPase activating protein neurofibromin.

Patients with NF1 have been felt to be at increased risk of a variety of GI tumors. A previous report reviewing the literature found that 34% of GI tract malignancies in patients with NF1 were GISTs [38]. NF1 associated GISTs are somewhat different than typical sporadic GISTs as NF1 associated GISTs typically have neither mutated *KIT* nor *PDGFRA* genes [44,45]. One series, nevertheless, reported that a small number of NF1 patients did have *KIT* and *PDGFRA* mutations [46].

Since the majority of GISTs associated with NF1 mutations do not have *c-KIT* or *PDGFRA* mutations, NF1 associated GISTs may have alternative pathogenesis which may result in different clinical outcomes. The majority of the tumors from patients in the series by Miettinen *et al.* 2006 had small, mitotically inactive tumors, and the majority of patients in this series with long-term follow-up had good prognosis [44]. Whether NF1 associated GISTs arise from a completely KIT independent process or whether the mutated neurofibromin protein indirectly activates KIT pathways carries significant clinical implications. Therapies that directly target KIT such as imatinib may not be effective in patients with NF1 associated GIST. Despite the lack of *KIT* mutations in most cases of NF1 GIST, one case report indicated disease stabilization with sunitinib [47].

## Succinate dehydrogenase mutations

Succinate dehydrogenase (SDH) is an enzyme localized to the inner mitochondrial membrane and is integral to cellular respiration by participating in both the citric acid cycle and the electron transport chain. SDH is composed of four subunits (A-D), and mutations in the SDH genes encoding each subunit have been associated with various human diseases [48]. Not surprisingly, a number of these diseases involve disordered mitochondrial respiration, resulting in severe metabolic and neurologic dysfunction.

In addition to SDH’s critical role in cellular respiration, SDH is believed to function as a tumor suppressor. Mutations in SDH subunits B (SDHB), C (SDHC), and D (SDHD), in particular, have been associated with familial cancer predisposition syndromes with affected individuals at increased risk for the development of paragangliomas and pheochromocytomas [10]. In 2002, 12 individuals from five unrelated families were found to have developed paragangliomas and GISTs, and the Carney-Stratakis Syndrome (CSS) was described [39].

CSS appears to be an autosomal dominant syndrome with incomplete penetrance characterized by the development of paraganglioma, GIST, or both. CSS has a variable phenotypic expression as demonstrated by a report of monozygotic twins with CSS where one developed a paraganglioma and the other GIST [49]. To better characterize the germline mutations present in patients with CSS, genetic sequencing was performed for six individuals in six unrelated families, and mutations in *SDHB, SDHC,* and *SDHD,* were identified [40]. Differing from most sporadic GISTs, no mutations in KIT or PDGFRA were seen.

Additional recent work has confirmed the important role SDH plays in the pathogenesis of GISTs. A study of 34 GIST patients without *KIT* or *PDGFRA* mutations (WT GIST) revealed that four patients (12%) had *SDH* germline mutations, even in the absence of a family or personal history of paragangliomas. Further, even in patients without germline *SDH* mutations, patients with WT GISTs had complete loss or markedly reduced SDHB protein expression compared to patients with KIT mutant GIST. This further suggests the important role of SDH in the pathogenesis of GIST, in addition to cases where a germline mutation in *SDH* is found [50]. The finding of absent or low levels of SDH protein in patients with CSS was confirmed by a study showing that none of the four patients with CSS had positive immunohistochemical staining for SDHB, even though only one was found to have a germline mutation. This contrasted starkly with KIT and PDGFRA mutant GIST samples which all stained strongly for SDHB [12]. Though mutations in *SDHB, SDHC,* and *SDHD*, have been described most thoroughly, recently two young patients with GIST were found to have detectable mutations in SDHA, representing the first described cases of SDHA inactivation in GIST [51].

## Deletions in chromosome 1 involving succinate dehydrogenase C

Due to associations between SDH mutations and patients with paraganglioma and GIST syndromes, the presence of SDH mutations was evaluated in patients with Carney Triad (CT), a similar, though distinct, syndrome from CSS [52]. CT is felt to be non-hereditary and consists of having at least two of the triad of paraganglioma, GIST, and pulmonary chordoma. CT was first described in 1977 when Carney and colleagues reported seven unrelated women with the triad [14]. In 2007, comparative genomic hybridization studies were performed on 41 tumor samples from 37 patients with CT, and though no tumors had coding sequence mutations of the investigated *SDH* genes, a number of DNA copy changes were seen [52]. Particularly, deletions of chromosome 1p and 1q12-q21, the site of the *SDHC* gene, were found. Interestingly, other classic mutations associated with GIST such as *KIT* and *PDGFRA* were not found in these tumor specimens from patients with CT. This finding is consistent with results from other studies [15,53], raising the possibility that GISTs in CT arise from alternative pathologic mechanisms from most sporadic GISTs.

Consistent with an alternative genetic and possible pathologic mechanism of tumorigenesis, GISTs in CT behave differently clinically from most sporadic GISTs as they affect young women and have been associated with frequent lymph node metastasis, multifocality, and unpredictable behavior [20]. In a review published in 1999, Carney described a higher rate of gastric GIST and metastatic disease at presentation [29]. How deletions of chromosome 1p and 1q (in the region of the *SDHC* gene) contribute to the alternative clinical behavior of GISTs associated with CT remains to be explained.

## Treatment of patients with inherited GIST

Although various germline mutations have been associated with heterogeneous clinical syndromes, no data currently exist that inherited GIST should be treated differently from its sporadic counterpart. Nevertheless, on a theoretical basis, increasing knowledge of the various germline mutations associated with hereditary GIST suggests that different clinical approaches may ultimately show increased benefit. For example, when GIST is present in patients with paragangliomas (Carney-Stratakis Syndrome), due to the lack of mutations in *KIT and PDGFRA* (WT GIST), these tumors theoretically may be less sensitive to imatinib. This may also be true for inherited GIST associated with *NF1* mutations as *KIT* mutations are only found in small proportions of NF1 GIST. Clinical data are not yet available to address these theoretical speculations.

Inherited GIST associated with CSS has been shown to be related to deficits in SDH, and improved understanding of the mechanisms surrounding SDH regulation may lead to future therapeutic approaches. Unfortunately, patients with advanced WT GIST when treated with imatinib had decreased objective response, time to tumor progression, and overall survival compared to patients with KIT exon 11 mutations [54]. Mutations in SDH and NF1 may explain the non-KIT mediated pathogenesis in patients with WT GIST, and patients with inherited SDH mutations, and possibly NF1 mutations, may ultimately benefit from alternative targeted treatment. The relationship between SDH and NF1 to GIST pathogenesis will first need to be further clarified.

Patients whose GISTs are characterized by a deficiency in SDHB by immunohistochemistry have been described to have a somewhat different clinical course from the majority of GIST patients. Specifically, one study found that deficiency of SDHB was associated with a female predominance, gastric primary location, lymph node involvement, and similar morphology to GIST arising in pediatric patients [55]. Since these patients’ GISTs followed a more indolent course, SDHB deficient tumors may ultimately need to be managed differently.

Sunitinib may be particularly helpful for patients with GIST who develop resistance or intolerance to imatinib. A study involving 97 patients with metastatic, imatinib-resistant/intolerant GIST, demonstrated the particular efficacy of sunitinib in patients with primary exon 9 and WT GIST [56,57].

## Conclusions

Gastrointestinal stromal tumors are the most common mesenchymal tumor arising in the GI tract, and therapy targeting the molecular abnormalities involved in GIST pathogenesis has been a remarkable success in solid tumor oncology. Improved recognition of the associated clinical features of patients with inherited GIST likely to harbor germline mutations will lead to appropriate genetics evaluation and may influence family members wishing to evaluate their risk. Unlike other cancer predisposition syndromes such as Lynch Syndrome, some of the germline mutations that result in inherited GIST, such as mutations involving *KIT* and *PDGFR* genes, produce abnormal proteins that can be inhibited by currently available targeted therapy. Increasing knowledge of novel inherited germline mutations predisposing to GIST, particularly wild-type inherited GIST, may lead to improved understanding of perturbed molecular pathways in sporadic GIST as well. For instance, the recognition of genetic abnormalities in *SDH* in GISTs that are not associated with KIT and PDGFRA mutations introduces a new avenue of research that may have relevance for sporadic disease.

## Abbreviations

CSS: Carney-Stratakis syndrome; CT: Carney triad; GIST: Gastrointestinal stromal tumor; NF1: Neurofibromatosis-1; NF2: Neurofibromatosis-2; PDGFRA: Platelet derived growth factor receptor-alpha; SEER: Surveillance epidemiology and end results; SDH: Succinate dehydrogenase; WT: Wild type.

## Competing interests

The authors declare that they have no competing interests.

## Authors’ contributions

MP collected the references and wrote the first draft of the manuscript. MR revised the manuscript and provided intellectual guidance in support of the content of this project. Both authors read and approved the final manuscript.

## Authors’ information

MP is a medical oncology fellow in the Melanoma/Sarcoma Oncology Service at Memorial Sloan-Kettering Cancer Center. MR is the clinic director of the Clinical Genetics Service at Memorial Sloan-Kettering Cancer Center and a member of the Breast Cancer Medicine Service.
